# Comparison of S-Detect and thyroid imaging reporting and data system classifications in the diagnosis of cytologically indeterminate thyroid nodules

**DOI:** 10.3389/fendo.2023.1098031

**Published:** 2023-01-24

**Authors:** Ling Zhou, Lin-lin Zheng, Chuan-ju Zhang, Hong-fen Wei, Li-long Xu, Mu-rui Zhang, Qiang Li, Gao-fei He, Edem Prince Ghamor-Amegavi, Shi-yan Li

**Affiliations:** ^1^ Sir Run Run Shaw Hospital, School of Medicine, Zhejiang University, Hangzhou, China; ^2^ Second Affiliated Hospital, School of Medicine, Zhejiang University, Hangzhou, China

**Keywords:** computer-aided diagnosis, thyroid imaging reporting and data system, ultrasound, diagnosis, cytologically indeterminate thyroid nodule

## Abstract

**Purpose:**

The aim of this study was to investigate the value of S-Detect for predicting the malignant risk of cytologically indeterminate thyroid nodules (CITNs).

**Methods:**

The preoperative prediction of 159 CITNs (Bethesda III, IV and V) were performed using S-Detect, Thyroid Imaging Reporting and Data System of American College of Radiology (ACR TI-RADS) and Chinese TI-RADS (C-TIRADS). First, Linear-by-Linear Association test and chi-square test were used to analyze the malignant risk of CITNs. McNemar’s test and receiver operating characteristic curve were used to compare the diagnostic efficacy of S-Detect and the two TI-RADS classifications for CITNs. In addition, the McNemar’s test was used to compare the diagnostic accuracy of the above three methods for different pathological types of nodules.

**Results:**

The maximum diameter of the benign nodules was significantly larger than that of malignant nodules [0.88(0.57-1.42) vs 0.57(0.46-0.81), *P*=0.002]. The risk of malignant CITNs in Bethesda system and the two TI-RADS classifications increased with grade (all *P* for trend<0.001). In all the enrolled CITNs, the diagnostic results of S-Detect were significantly different from those of ACR TI-RADS and C-TIRADS, respectively (*P*=0.021 and *P*=0.007). The sensitivity and accuracy of S-Detect [95.9%(90.1%-98.5%) and 88.1%(81.7%-92.5%)] were higher than those of ACR TI-RADS [87.6%(80.1%-92.7%) and 81.8%(74.7%-87.3%)] (*P*=0.006 and *P*=0.021) and C-TIRADS [84.3%(76.3%-90.0%) and 78.6%(71.3%-84.5%)] (*P*=0.001 and *P*=0.001). Moreover, the negative predictive value and the area under curve value of S-Detect [82.8% (63.5%-93.5%) and 0.795%(0.724%-0.855%)] was higher than that of C-TIRADS [54.8%(38.8%-69.8%) and 0.724%(0.648%-0.792%] (*P*=0.024 and *P*=0.035). However, the specificity and positive predictive value of S-Detect were similar to those of ACR TI-RADS (*P*=1.000 and *P*=0.154) and C-TIRADS (*P*=1.000 and *P*=0.072). There was no significant difference in all the evaluated indicators between ACR TI-RADS and C-TIRADS (all *P*>0.05). The diagnostic accuracy of S-Detect (97.4%) for papillary thyroid carcinoma (PTC) was higher than that of ACR TI-RADS (90.4%) and C-TIRADS (87.8%) (*P*=0.021 and *P*=0.003).

**Conclusion:**

The diagnostic performance of S-Detect in differentiating CITNs was similar to ACR TI-RADS and superior to C-TIRADS, especially for PTC.

## Introduction

Thyroid cancer is the most frequent endocrine cancer worldwide and ranks the ninth for incidence among all malignancies (1). The incidence of thyroid cancer has increased over the past few decades in many countries (1, 2). Nowadays, the thyroid nodule (TN) has become one of the most common causes of seeking medical advice in daily clinical practice. Ultrasonography (US) has been used as the preferred imaging method for detecting and diagnosing TNs in the routine clinical setting (3). The increase of incidence of TN is likely caused to increased imaging usage, which increases from 4-7% by palpation to 19-67% by US in asymptomatic population (4). In addition, US could be used to reveal information about the margin, shape, composition and echogenicity, and to make a differential diagnosis of TNs (3, 4). Many systems have been established to predict the malignant risk of TNs based on US characteristics, such as Thyroid Imaging Reporting and Data System of American College of Radiology (ACR TI-RADS) (5) and Chinese TI-RADS (C-TIRADS) (6), and are useful for describing thyroid disease (7).

At present, US-guided fine-needle aspiration (FNA) is considered as the standard workup for judging benign or malignant TNs preoperatively (8), but FNA still can not reliably give definitive results in about 10-20% of TNs (9–11). The results of FNA are presented by using the Bethesda System for Reporting Thyroid Cytopathology (TBSRTC) (12). There are six categories in this system. Bethesda I (Nondiagnostic or unsatisfactory) TNs are usually required repeated FNA. Bethesda II (Benign) and VI (Malignant) TNs will be managed without any confusion. For TNs with Bethesda IV (Follicular neoplasm or Suspicious for a follicular neoplasm) and V (Suspicious for malignancy), preoperative molecular testing (Bethesda IV) or surgical procedure (Bethesda V) is suggested (12). But there is still the possibility that postoperative specimens are benign lesions, leading to over-diagnosis and over-treatment (13). The nature of TNs with Bethesda III (Atypia of undetermined significance or Follicular lesion of undetermined significance) is also uncertain, which even accounts for about 15% at the first FNA (10, 11). Therefore, the above three categories (Bethesda III, IV and IV) of TNs can be considered as the cytologically indeterminate TNs (CITNs), which lead to confusion in clinical practice. As benign TNs have little effects on patient’s health and could be managed conservatively, efforts to better select candidates for operation are necessary.

For further evaluation of CITNs, repeated FNA, molecular testing, or diagnostic thyroidectomy may be arranged for the patient. However, these also remain a challenge. There are 20-28% patients with Bethesda III category presented as CITNs again on the second FNA (14). The risk of malignancy in nodules with Bethesda III category sent directly to surgery is only 15.7% (15). Although BRAF mutation testing could be used as a supplement to routine cytology, its value in improving the preoperative diagnostic rate for malignancy is limited (16). As mentioned above, the findings of US could be used to predict malignancy, especially when cytology is not conclusive. The predicted value of several stratified systems that are based on US features (such as ACR TI-RADS and C-TIRADS) for assessing CITNs has been investigated in previous studies (17, 18). However, these classifications might be intricate for inexperienced physicians (19).

In recent years, computer-aided diagnosis system using artificial intelligence (AI-CADS) based on US image analysis techniques has been developed and introduced to the clinical application of US medicine (20–23). Its utility has been preliminarily verified in the detection of breast and thyroid malignant nodules with satisfactory results (21, 22). Many previous studies have shown that AI-CADS for the classification of TNs can achieve accuracy comparable to senior sonologists, reduce operator dependency, and provide second advice in imaging diagnosis (22–24). As a kind of AI-CADS, S-Detect software (Samsung Ultrasound RS80A, Samsung Medison Co. Ltd., Seoul, South Korea) is an image-analytic program developed on the basis of statistical data mining algorithm, which can directly judge the nature of TNs in ultrasound images and achieve the consistency of the US features of the same nodules (25, 26). Previous studies have shown that S-Detect has reliable diagnostic performance for TNs with Bethesda II and VI categories (26–28). However, as far as we know, there is no relevant study on the performance of S-Detect in differentiating CITNs.

Therefore, the purpose of this study was to investigate the value of S-Detect based on US images for predicting the malignant risk of CITNs by comparing with the two TI-RADS classifications in CITNs.

## Materials and methods

This retrospective study was performed in line with the principles of the Declaration of Helsinki. Approval was approved by the Ethics Committee of our institution (No. 20201217-38). Informed consent was obtained from each of the participants prior to the data collection for scientific research and potential publication of their anonymized images.

### Patients

Between January 2020 and December 2021, the imaging data of 536 CITNs from 497 patients who underwent thyroidectomy in our hospital were collected retrospectively. Excluding cases without thyroidectomy (358 CITNs) and incomplete imaging data (19 CITNs), 145 patients with 159 CITNs were finally enrolled (30 males and 115 females) in the study. The mean age was 46.09 ± 12.09 years (range from 18 to 76 years) and the mean size of the maximum diameter of the TNs was 0.97 ± 0.97 cm (range from 0.24 to 6.63 cm).

The inclusion criteria were as follows: (1) the result of FNA was indeterminate (Bethesda III, IV and V categories); (2) underwent thyroidectomy in our hospital and with clear postoperative pathological diagnosis (confirmed by two experienced pathologists); (3) the US examination and S-Detect were performed before FNA, and the time interval between US and surgery was less than 3 weeks. The exclusion criteria were as follows: (1) the result of FNA was Bethesda I, II or VI category; (2) TNs with repeated FNA; (3) incomplete ultrasound images and clinical date.

### S-Detect software for TNs

In this study, a US machine (Samsung Ultrasound RS80A, Samsung Medison Co. Ltd., Seoul, South Korea) with a 3-12 MHz linear array probe was used. The S-Detect was integrated into the US system. The sonologists opened the S-Detect interface and entered the optimal preset for thyroid. First, longitudinal and transverse scans were performed to the suspected TNs, and the maximum diameter of the TNs were taken as the representative images for S-Detect and two TI-RADS classifications. The contours of the TNs could then be drawn automatically by the S-Detect software or manually when needed. S-Detect evaluated US characteristics of TNs, including composition, echogenicity, orientation, margin, shape and spongiform status, and gave two diagnostic opinions: possibly benign and possibly malignant. Finally, all image data were stored. The US images were collected by two sonologists with 6 and 9 years of experience in the thyroid US who were blinded to the pathology results.

### ACR TI-RADS and C-TIRADS

According to the 2017 ACR TI-RADS publication (5), each US feature received point(s) and the individual points summed up. The classification was arranged based on the total score (TR1: 0 point; TR2: 2 points; TR3: 3 points; TR4: 4-6 points; TR5: ≥7 points). US characteristics include composition, echogenicity of solid components, shape, margin and calcification (**Table 1**).

**Table 1:** Thyroid Imaging Reporting and Data System of American College of Radiology (5).

Ultrasound features		Point
Composition (chose 1)	Cystic or almost completely cystic	0
	Spongiform	0
	Mixed cystic and solid	+1
	Solid or almost completely solid	+2
Echogenicity (chose 1)	anechoic	0
	Hyperechoic or isoechoic	+1
	Hypoechoic	+2
	Very hypoechoic	+3
Shape (chose 1)	Wider than tall	0
	Taller than wide	+3
Margin (chose 1)	Smooth	0
	Ill-defined	0
	Lobulated/irregular	+2
	Extra-thyroidal extension	+3
Echogenic foci (chose all that apply)	None or large comet tall artefact	0
	Macrocalcifications	+1
	Peripheral/rim calcifications	+2
	Punctate echogenic foci	+3

The TNs were assessed according to C-TIRADS proposed by Zhou (6). The category was established by counting US features, including solid composition, microcalcifications, markedly hypoechoic, irregular margin/ill-defined or extrathyroidal extension, vertical orientation and Comet tail artifacts (subtraction point). The classification ranged from TR1 to TR6 (TR1: no nudule; TR2: -1 point; TR3: 0 point; TR4A: 1 point; TR4B: 2 points; TR4C: 3-4 points; TR5: 5 points; TR6: biopsy proved malignant) (**Table 2**).

**Table 2:** Chinese thyroid imaging reporting and data system (6).

Ultrasound features	Counting value
Solid composition	+1
Microcalcifications	+1
Markedly hypoechoic	+1
Irregular margin/ill-defined or extrathyroidal extension	+1
Vertical orientation	+1
Negative US features	
Comet tail artifacts	-1
C-TIRADS	Score
1, no nodule	Not available
2, benign	-1 Point
3, probably benign	0 Point
4A, low suspicion	1 Point
4B, moderate suspicion	2 Points
4C, high suspicion	3 to 4 Points
5, highly suggestive of malignancy	5 Points
6, biopsy proved malignant	Not available

In this study, the postoperative pathology of TNs was taken as the gold standard, and the cut-off values were obtained by receiver operating characteristic (ROC) curves analysis of the results of the two TI-RADS classifications.

### Image interpretation

Firstly, all indicators of ACR TI-RADS and C-TIRADS were listed separately and arranged uniformly (**Table 3**). Then, two sonologists with more than 10 years of experience in thyroid US assessed the enrolled images of CITNs independently and ticked the indicators in the images respectively. If no consensus was reached, arbitration from another sonologist (with more than 20 years of experience in thyroid US) was performed. Finally, a sonologist with more than 3 years of experience accumulated the scores according to the indicators, and obtained ACR TI-RADS and C-TIRADS results of each image respectively. The above three reviewers were blinded to the findings of each other, the results of S-Detect, the pathological results and other clinical information of the CITNs.

**Table 3:** Summary list of ultrasound features for TI-RADS classifications.

Ultrasound features (Please tick in the below box.)			
Composition (chose 1)			
□ Cystic or almost completely cystic	□ Spongiform	□ Mixed cystic and solid	□ Solid or almost completely solid
Echogenicity (chose 1)			
□ anechoic	□ Hyperechoic or isoechoic	□ Hypoechoic	□ Very hypoechoic
Shape (chose 1)			
□ Wider than tall		□ Taller than wide	
Margin (chose 1)			
□ Smooth	□ Ill-defined	□ Lobulated/irregular	□ Extra-thyroidal extension
Echogenic foci (chose all that apply)			
□ None or large comet tall artefact	□ Macrocalcifications	□ Peripheral/rim calcifications	□ Punctate echogenic foci

TI-RADS, Thyroid Imaging Reporting and Data System.

### Pathology

All of the cytological and histological findings of the enrolled CITNs were reviewed and confirmed by two pathologists (with more than 10 years experience of cytopathology or histopathology respectively) independently, and they were blinded to the findings of each other. Another pathologist with more than 15 years experience made the final decision if no consensus was reached. To ensure the corresponding relationship between pathology and US image, the size and location of each CITN was compared, such as left or right lobe or isthmus; superior pole or inferior pole or middle; ventral or dorsal or middle; and interior or middle or lateral.

### Statistical analysis

All statistical analyses were performed using SPSS v19.0 (SPSS Inc, Chicago, IL) and MedCalc v15.0 (MedCalc Software, Ostend, Belgium). If the variables were quantitative and normal, the mean ± standard deviation (SD) was used for statistical description, and the median with interquartile range (IQR) was used for the non-normal variables. Counting data is presented as numbers and percentage. The normal distribution data were compared between groups using an independent sample *t*-test. For non-normal distribution data, differences were analyzed using a Mann-Whitney *U* test. Chi-square test or McNemar’s test was used to compare the differences between groups for counting data. Linear-by-Linear Association test was used to analyze the linear trend of the malignant risk of CITNs. The ROC curve was drawn according to the histological results as the reference standard to find the best diagnostic cutoff value for the two TI-RADS classifications. In addition, the area under curve (AUC) of ROC curve was used to compare the diagnostic efficacy between S-Detect and the two TI-RADS classifications by Delong test (29). *P* values less than 0.05 were considered as statistically significant.

## Results

### Clinicopathological characteristics of enrolled nodules

Totally, 159 CITNs from 145 patients were enrolled in this study (**Table 4**). Postoperative pathology revealed 38 benign TNs and 121 malignant TNs. The maximum diameter of benign nodules was significantly larger than that of malignant nodules (*P*=0.002), while there was no significant difference between benign and malignant nodules in terms of age and gender (*P*=0.092 and *P*=0.167).

**Table 4:** Clinicopathological characteristics of the cytologically Indeterminate thyroid nodules.

Parameter	Pathological findings		*P*-value
	Benign (n = 38)	Malignant (n = 121)	
Age (years), Mean ± SD	51.87 ± 9.63	44.28 ± 12.25	0.092^1^
Sex (number of patients)			0.167^2^
Male	4(13.3)	26(86.7%)	
Female	29(25.2%)	86(74.8%)	
Maximum nodule size (cm), Median (IQR)	0.88 (0.57-1.42)	0.57 (0.46-0.81)	0.002^3^
Bethesda category			
III (n)	27 (71.1%)	33 (27.3%)	<0.001^2^
IV (n)	4 (10.5%)	8 (6.6%)	
V (n)	7 (18.4%)	80 (66.1%)	
S-Detect			<0.001^2^
Benign (n)	24 (63.2%)	5 (4.1%)	
Malignant (n)	14 (36.8%)	116 (95.9%)	
ACR TI-RADS			<0.001^2^
Benign (n)	24 (63.2%)	15 (12.4%)	
Malignant (n)	14 (36.8%)	106 (87.6%)	
C-TIRADS			<0.001^2^
Benign (n)	23 (60.5%)	19 (15.7%)	
Malignant (n)	15 (39.5%)	102 (84.3%)	

ACR TI-RADS, Thyroid Imaging Reporting and Data System of American College of Radiology; C-TIRADS, Chinese TI-RADS; cm, Centimeter; IQR, interquartile range; n: Number; SD, standard deviation; ^1^ Independent sample t-test; ^2^ Chi-square test; ^3^ Mann-Whitney U test.

According to ROC curve analysis, the ACR TI-RADS took TR4 as the cut-off value, and the nodules above TR4 were considered as possible malignant. The C-TIRADS took TR4B as the cut-off value, and the nodules above TR4B were also considered as possible malignant. ACR TI-RADS detected 39 benign nodules and 120 malignant nodules. C-TIRADS detected 42 benign nodules and 117 malignant nodules. S-Detect detected 29 benign nodules and 130 malignant nodules. The diagnostic results of the above three methods were statistically different from the pathological results (all *P*<0.001).

### Malignancy risk of the CITNs in different categories

The malignant risk of CITNs in Bethesda system and different TI-RADS classifications increased with classification grade (**Table 5**). The malignant risk of nodules among Bethesda III, IV and V categories was 55.0% (33/60), 66.7% (8/12) and 92.0% (80/87), respectively (*P* for trend < 0.001). The risk of malignancy among ACR TI-RADS 3, 4 and 5 was 0% (0/0), 42.9% (15/35) and 88.3% (106/120), respectively (*P* for trend < 0.001). And the risk of malignancy in C-TIRADS 3, 4A, 4B, 4C and 5 was 0% (0/0), 7.7% (1/13), 62.1% (18/29), 85.4% (82/96) and 95.2% (21/21) (*P* for trend < 0.001).

**Table 5:** Malignancy risk of the cytologically indeterminate thyroid nodules in different categories.

Category	Classification level							*P*-value^1^
Bethesda category	III		IV			V		
Benign	27		4			7		<0.001
Malignant	33		8			80		
Risk of Malignancy (%)	55.0		66.7			92.0		
*P* for trend^2^	<0.001							
ACR TI-RADS	3	4					5	
Benign	4	20					14	<0.001
Malignant	0	15					106	
Risk of Malignancy (%)	0	42.9					88.3	
*P* for trend^2^	<0.001							
C-TIRADS	3	4A		4B	4C		5	
Benign	0	12		11	14		1	<0.001
Malignant	0	1		18	82		20	
Risk of Malignancy (%)	0	7.7		62.1	85.4		95.2	
*P* for trend^2^	<0.001							

ACR TI-RADS, Thyroid Imaging Reporting and Data System of American College of Radiology; C-TIRADS, Chinese TI-RADS. ^1^ Chi-square test, ^2^ Linear-by-Linear Association test.

### Comparison of diagnostic performance between S-Detect and the two TI-RADS classifications for differentiating CITNs

In all the CITNs, the diagnostic results of S-Detect were significantly different from those of ACR TI-RADS and C-TIRADS, respectively (*P*=0.021 and *P*=0.007). However, in each of Bethesda categories, such as Bethesda III, IV, or V, there was no significant difference between the S-Detect and the two TI-RADS classifications (all *P*>0.05) (**Table 6**).

**Table 6:** Differences between S-Detect and the two TI-RADS classifications.

Bethesda category	Variable	*P*-value^1^
Bethesda III, IV and V	S-Detect vs ACR TI-RADS	0.021
	S-Detect vs C-TIRADS	0.007
Bethesda III	S-Detect vs ACR TI-RADS	0.063
	S-Detect vs C-TIRADS	0.375
Bethesda IV	S-Detect vs ACR TI-RADS	0.500
	S-Detect vs C-TIRADS	0.250
Bethesda V	S-Detect vs ACR TI-RADS	0.508
	S-Detect vs C-TIRADS	0.092

TI-RADS, Thyroid Imaging Reporting and Data System; ACR TI-RADS, American College of Radiology TI-RADS; C-TIRADS, Chinese TI-RADS, n: Number. ^1^ McNemar’s test.

**Figure 1:**
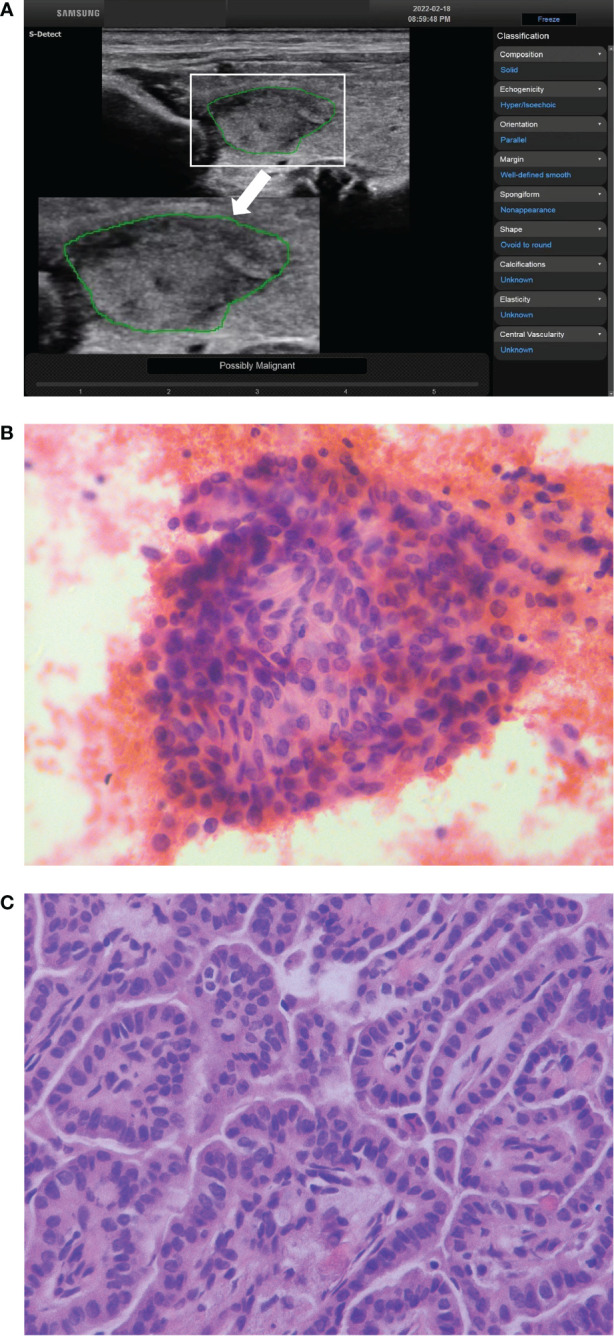
The ultrasound features of a thyroid nodule in a 28-year-old woman were solid, hypoechoic, wider than tall, smooth margin and without echogenic foci. S-Detect indicated “Possibly Malignant” **(A)**. The ACR TI-RADS was TR4 with 4 points, and the C-TIRADS was TR4A with 1 point. It was considered to be a benign nodule. The nodule was classified as Bethesda V category after fine-needle aspiration **(B)** (HE staining; magnification ×400). And the final pathological result showed papillary thyroid carcinoma **(C)** (HE staining; magnification ×400).

**Figure 2:**
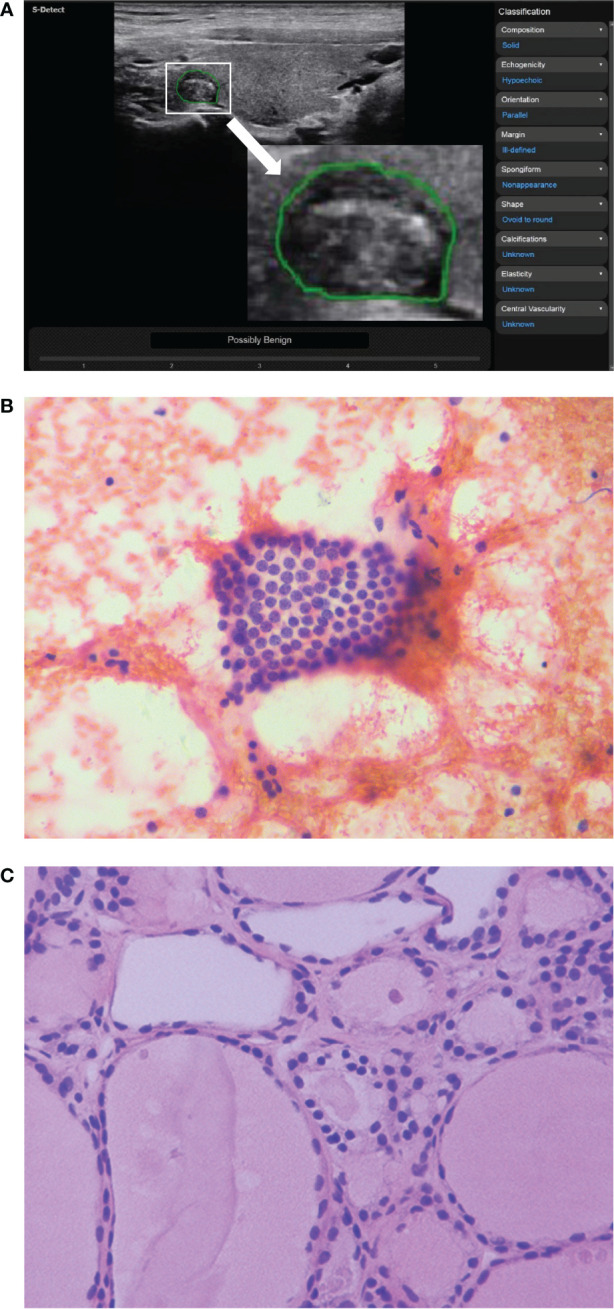
The ultrasound features of a thyroid nodule in a 32-year-old woman were solid, hypoechoic, wider than tall, ill-defined margin and punctate echogenic foci. S-Detect indicated “Possibly Benign” **(A)**. However, the ACR TI-RADS was TR5 with 7 points, and the C-TIRADS was TR4C with 3 points. It was considered to be a malignant nodule. The nodule was classified as Bethesda III category after fine-needle aspiration **(B)** (HE staining; magnification ×400). And the final pathological result suggested nodular goiter **(C)** (HE staining; magnification ×400).

In all the enrolled CITNs, the sensitivity, specificity, positive predictive value (PPV), negative predictive value (NPV), and accuracy for diagnosing malignant TNs were 95.9%, 63.2%, 89.2%, 82.8%, and 88.1% for S-Detect, 87.6%, 63.2%, 88.3%, 61.5%, and 81.8% for ACR TI-RADS, and 84.3%, 60.5%, 87.2%, 54.8% and 78.6% for C-TIRADS, respectively (**Table 7**). The sensitivity and accuracy of S-Detect were higher than those of ACR TI-RADS (*P*=0.006 and *P*=0.021) and C-TIRADS (*P*=0.001 and *P*=0.001). And the NPV of S-Detect was higher than that of C-TIRADS (*P*=0.024). While specificity and PPV of S-Detect were similar to those of ACR TI-RADS (*P*=1.000 and *P*=0.154) and C-TIRADS (*P*=1.000 and *P*=0.072). Moreover, there was no significant difference in all evaluated indexes between ACR TI-RADS and C-TIRADS (all *P*>0.05) (**Figures 1** and **2**).

**Table 7:** Comparison of the diagnostic performance of S-Detect and the two TI-RADS classifications.

Diagnostic means	Sensitivity (%) (95% CI)	Specificity (%) (95% CI)	PPV (%) (95% CI)	NPV (%) (95% CI)	Accuracy (%) (95% CI)	AUC (95% CI)
S-Detect	95.9 (90.1-98.5)	63.2 (46.0-77.7)	89.2 (82.3-93.8)	82.8 (63.5-93.5)	88.1 (81.7-92.5)	0.795 (0.724-0.855)
ACR TI-RADS	87.6 (80.1-92.7)	63.2 (46.0-77.7)	88.3 (80.9-93.2)	61.5 (44.7-76.2)	81.8 (74.7-87.3)	0.754 (0.679-0.819)
C-TIRADS	84.3 (76.3-90.0)	60.5 (43.5-75.5)	87.2 (79.4-92.4)	54.8 (38.8-69.8)	78.6 (71.3-84.5)	0.724 (0.648-0.792)
*P* value^1^	0.006	1.000	0.154	0.064	0.021	0.169
*P* value^2^	0.001	1.000	0.072	0.024	0.001	0.035
*P* value^3^	0.219	1.000	0.736	0.736	0.125	0.073

ACR TI-RADS, Thyroid Imaging Reporting and Data System of American College of Radiology; AUC, Area under curve; CI, Confidence interval; C-TIRADS, Chinese TI-RADS NPV, Negative predictive value; PPV, Positive predictive value. ^1^ S-Detect vs ACR TI-RADS; ^2^ S-Detect vs C-TIRADS; ^3^ ACR TI-RADS vs C-TIRADS. McNemar’s test was used for all P values.

**Figure 3:**
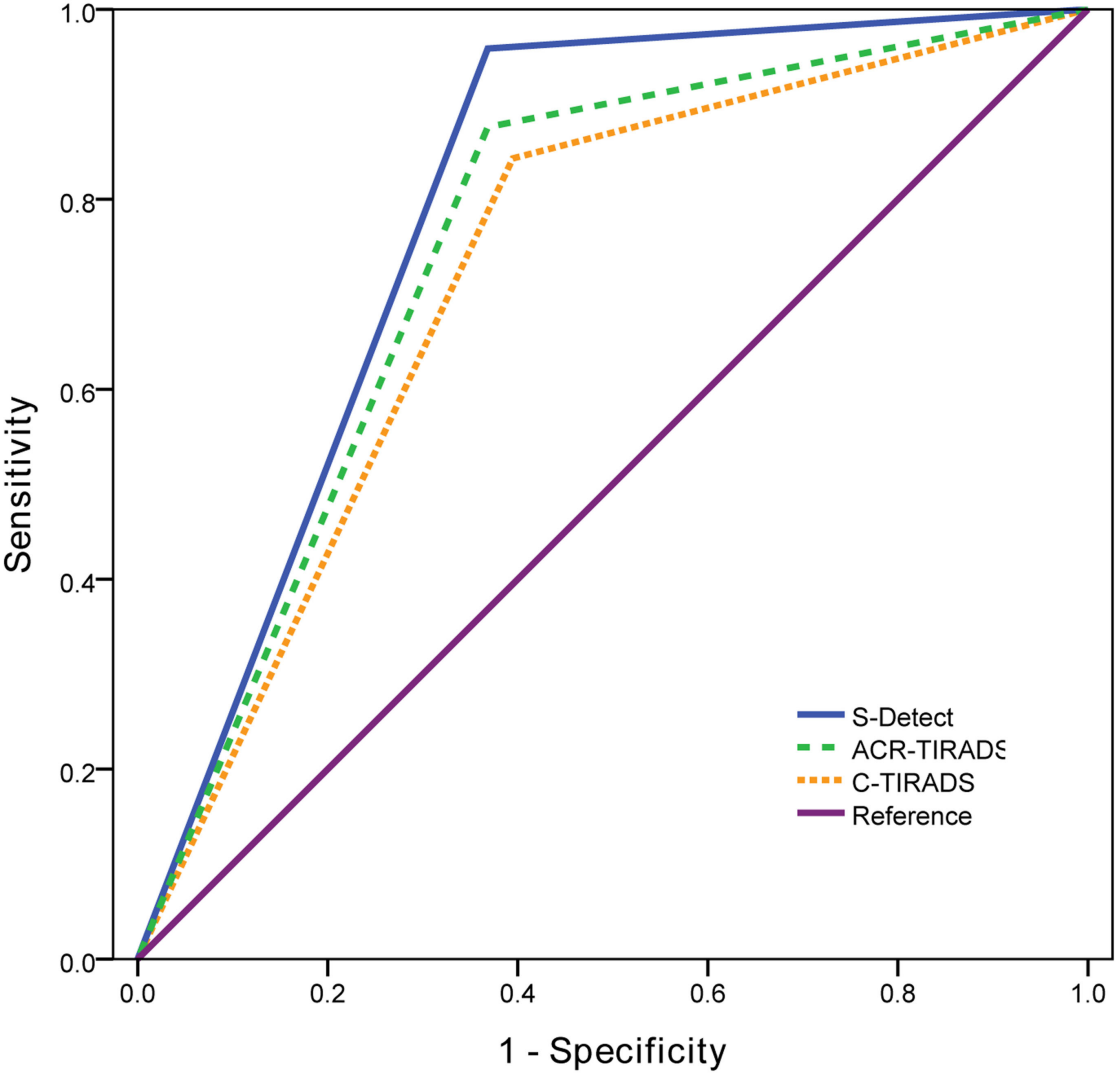
The receiver operating characteristic (ROC) curves of S-Detect and two TI-RADS classifications.

ROC curves were drawn for S-Detect and the two TI-RADS classifications (**Figure 3**). The AUC value of S-Detect [0.795, 95% confidence interval (CI): 0.724-0.855] was higher than C-TIRADS (0.724, 95% CI: 0.648-0.792) (*Z*=2.111, *P*=0.035). However, the AUC value between S-Detect and ACR TI-RADS (0.754, 95% CI: 0.679-0.819) (*Z*=1.375, *P*=0.169) was not statistically significant difference, nor was the AUC between ACR TI-RADS and C-TIRADS (*Z*=1.793, *P*=0.073).

### Comparison of diagnostic efficacy of S-Detect and the two TI-RADS classifications in different pathological types

Seven pathological types were present in 38 benign nodules, including nodular goiter (n=13), lymphocytic thyroiditis (n=6), follicular adenoma (n=10), subacute thyroiditis (n=2), thyroid fibro-hyaline nodule (n=5), focal fibroplasia (n=1), and granulomatous inflammation (n=1). Three pathological types were found in 121 malignant nodules, including papillary thyroid carcinoma (PTC) (n=115) and follicular thyroid carcinoma (FTC) (n=6) (**Table 8**). The diagnostic accuracy of S-Detect for PTC was higher than that of ACR TI-RADS and C-TIRADS (*P*=0.021, *P*=0.003, respectively), while for other pathological types, there was no significant difference in the diagnostic results of the three methods (all *P*>0.05).

**Table 8:** Final Pathology of the cytologically indeterminate thyroid nodules.

Postoperative pathology (n=159)	Accuracy			*P* ^1^	*P* ^2^
	S-Detect	ACR TI-RADS	C-TIRADS		
Benign (n=38)	24 (63.2%)	24 (63.2%)	23 (60.5%)		
Nodular goiter (n=13)	9 (69.2%)	8 (61.5%)	8 (61.5%)	1.000	1.000
Lymphocytic thyroiditis (n=6)	3 (50.0%)	4 (66.7%)	3 (50.0%)	1.000	1.000
Follicular adenoma (n=10)	8 (80.0%)	8 (80.0%)	8 (80.0%)	1.000	1.000
Subacute thyroiditis (n=2)	1 (50.0%)	1 (50.0%)	1 (50.0%)	1.000	1.000
Thyroid fibro-hyaline nodule (n=5)	2 (40.0%)	2 (40.0%)	2 (40.0%)	1.000	1.000
Focal fibrous tissue hyperplasia (n=1)	0 (0.0%)	0 (0.0%)	0 (0.0%)	/	/
Granulomatous inflammation (n=1)	1 (100.0%)	1 (100.0%)	1 (100.0%)	/	/
Malignant (n=121)	116 (95.9%)	106 (87.6%)	102 (84.3%)		
Papillary thyroid carcinoma (n=115)	112 (97.4%)	104 (90.4%)	101 (87.8%)	0.021	0.003
Follicular thyroid carcinoma (n=6)	4 (66.7%)	2 (33.3%)	1 (16.7%)	0.500	0.250

ACR TI-RADS, Thyroid Imaging Reporting and Data System of American College of Radiology; C-TIRADS, Chinese TI-RADS; n, Number. ^1^ S-Detect vs ACR TI-RADS, ^2^ S-Detect vs C-TIRADS. McNemar’s test was used for all P values.

## Discussion

At present, the main method to diagnose suspicious TNs is US-guided FNA combined with TBSRTC. However, CITN is a gray zone of cytological diagnosis and remains a challenge in clinical practice (30). For further diagnosis, S-Detect and the two TI-RADS classifications were used for preoperative assessment of CITNs in this study. S-Detect is a highly recognized US thyroid imaging platform based on AI-CADS. The system include two outputs: TI-RADS based on scoring and dichotomous predictions. The prediction is a independent diagnosis based on convolutional neural network deep learning techniques (31, 32). S-Detect has the ability to evaluate TNs in real time, which could reduce subjectivity to a certain extent (23, 24). ACR TI-RADS and C-TIRADS are widely used and have important value in determining the nature of nodules (5, 6). In this study, the diagnostic efficacy of the two TI-RADS classifications for CITNs was similar without significant difference.

### The diagnostic difference between S-Detect and the two TI-RADS classifications in each Bethesda category

In this study, the malignant risk for Bethesda III, IV, and V TNs increased with grade, similar to the ACR TI-RADS and C-TIRADS, but benign and malignant nodules were found in each category. In all the CITNs, there were significant differences between S-Detect and the two TI-RADS classifications. However, there was no significant difference in the diagnosis results of S-Detect and the two TI-RADS classifications in each category (Bethesda III, IV, and V). The nodules with Bethesda V had a high malignant risk, so it was understandable that S-Detect and the two TI-RADS classifications had similar diagnostic efficacy. For Bethesda III and IV nodules, the reason might be related to the low number of cases, especially for Bethesda IV nodules, which needed to be further confirmed in future studies. In addition, malignancy rates of Bethesda III, IV, and V in the study population were much higher than those of the general population (33), which maybe associated with bases on surgical cohorts selection bias.

### The diagnostic efficacy of S-Detect and the two TI-RADS classifications in the all enrolled CITNs

The diagnostic performance of S-Detect in discriminating CITNs was similar to ACR TI-RADS and superior to C-TIRADS. In terms of sensitivity and accuracy, S-Detect was higher than the two TI-RADS classifications. According to previous studies (34, 35), S-Detect analyzed and weighted US features of many malignant nodules to identify suspicious TNs, such as composition, echogenicity, shape, margin, calcification and extrathyroidal extension. So S-Detect was more detailed and objective in the identification process. While the malignant features judged by two TI-RADS classifications were relatively limited, which could not comprehensively and reliably predict the nature of TNs (5, 6, 36). Moreover, the interpretation process depends on visual observation and personal experience, which also affected the diagnostic efficiency (19). In terms of specificity and PPV, S-Detect needed to be improved, which was consistent with the results of many studies (21, 28, 37). This could be attributed to the three factors. First, some TI-RADS classifications related to the development of S-Detect software, such as Kwak TI-RADS, itself have high sensitivity and low specificity (26). Secondly, S-Detect is not accurate enough to recognize calcifications (22). For S-Detect, benign lesions with calcification are likely to show false-positive results, whereas, the smaller lesions without calcifications were likely to show false-negative results (38). Furthermore, in this study, all the enrolled TNs were suspected by US, the TI-RADS grade was relatively higher, and the number of benign nodules was relatively small, which could easily lead to biased results. However, some studies believed that S-Detect still had higher specificity and PPV compared with different TI-RADS classifications (27). Therefore, it is generally accepted that the diagnostic efficacy of S-Detect is different from that of different TI-RADS classifications. Of course, radiologists with different experience can also give different TI-RADS results for the same thyroid nodule. Chung et al. (28) believed that the diagnostic efficacy of S-Detect had obvious advantages for radiologists with less experience in the diagnosis of thyroid nodule.

### The diagnostic efficacy of S-Detect and the two TI-RADS classifications in different pathological types

PTC is the highest incidence of thyroid malignant tumors (1, 2), so the training sets of S-Detect are mainly based on the US features of PTC (39). Therefore, S-Detect has satisfactory diagnostic performance for PTC (40), which was also confirmed in this study. The diagnostic accuracy of S-Detect for PTC was significantly higher than that of the two TI-RADS classifications. However, for other malignant pathological types, such as FTC, there was no significant difference in the diagnostic results, which may be related to the limited number of cases, and it is necessary to increase the number of cases for further research in the future. Of course, the preoperative diagnosis of follicular thyroid neoplasm has been challenging. It is difficult to distinguish a benign or malignant TN based on cytological examination of follicular neoplasm (41). The presence of tumor capsule invasion and angioinvasion could be assessed only by postoperative histological pathology (42). Preoperative TI-RADS had been used to predict malignancy for Bethesda IV category TNs in previous studies (17, 18, 43), and the results showed that the risk of malignancy was 50.0% (18). This is similar to present study and indicated that the follicular neoplasm presented a confusing US characteristics. FTC could be found with a larger size, ovoid shape, and well-defined margins, which were regarded as benign features (44), so the diagnostic value of AI-CADS was also limited. Although the diagnostic accuracy of S-Detect in this study was 66.7%, which was higher than that of ACR TI-RADS (33.3%) and C-TIRADS (16.7%), there was no significant difference between the diagnostic results.

### The relationship between size and nature of CITNs

In addition, the maximum diameter of TNs may also be used as an auxiliary diagnostic method. This has been proven in some studies (40, 45). With the resolution of ultrasonic diagnostic instrument continuously improve and residents’ health consciousness enhancement, malignant nodules in small size can often be detected in the regular health examination, so as to obtain intervention treatment. Whereas benign nodules are generally not intervened, and have the opportunity to increase in size over time. So the maximum diameter of malignant nodules is often smaller than that of benign nodules (40), which is consistent with the results in this study. Of course, there are a small number of malignant nodules that are detected with large size, which is often associated with less differentiated nodules or patients who have not undergone a health examination for a long time.

## Limitations

There are several limitations in this study. Firstly, the images used in this study are static two-dimensional images, and sonographers may have missed some important information when analyzing TNs images. Secondly, the number of TNs with Bethesda III and IV categories was relatively small. Similarly, PTC was the mainly pathological type in this study, and the number of other pathological types was relatively short. These may cause sampling bias to a certain extent. Thirdly, the S-Detect used in this study focused on PTC mainly. There is relatively little training for other types of thyroid tumors. This may lead to poor diagnostic ability of S-Detect for other tumors.

## Conclusion

S-Detect based on US images could be used to predict malignant risk of CITNs after FNA. Although its performance cannot replace the pathological results, it was similar to ACR TI-RADS and superior to C-TIRADS in this study. Especially when the pathological type was PTC, S-Detect had the opportunity to become an alternative to TI-RADS.

## Data availability statement

The raw data supporting the conclusions of this article will be made available by the authors, without undue reservation.

## Ethics statement

The studies involving human participants were reviewed and approved by the Ethics Committee of Sir Run Run Shaw Hospital, School of Medicine, Zhejiang University. The patients/participants provided their written informed consent to participate in this study. Written informed consent was obtained from the individual(s) for the publication of any potentially identifiable images or data included in this article.

## Author contributions

LZ proposed the research ideas, designed the method and wrote the original draft. S-YL put forward some suggestions on the research ideas and reviewed and revised the paper. H-FW and L-LZ collected ultrasound images and obtained the diagnosis results of S-Detect. C-JZ, QL and L-LX were responsible for the interpretation of ultrasound image features. L-LZ counted the counting results of ultrasound image features. M-RZ was responsible for the diagnosis of pathological results. G-FH collected the patients’ clinical data. EG-A finally proofread the paper. All authors contributed to the article and approved the submitted version.
